# Recent Progress in Optical Chemical Sensors

**DOI:** 10.3390/s121216522

**Published:** 2012-11-29

**Authors:** Hummad Habib Qazi, Abu Bakar bin Mohammad, Muhammad Akram

**Affiliations:** 1 Infocomm Research Alliance (ICRA), Faculty of Electrical Engineering, Universiti Teknologi Malaysia, Skudai 81310, Johor, Malaysia; E-Mail: hqhummad3@live.utm.my; 2 Department of Chemistry, Faculty of Science, Universiti Teknologi Malaysia, Skudai 81310, Johor, Malaysia; E-Mail: sangra_utm@hotmail.com

**Keywords:** optical chemical sensors, fiber optic chemical sensors, integrated optical sensors, chemo-optical sensor

## Abstract

Optical chemical sensors have promoted escalating interest in the determination of various pollutants in the environment, which are creating toxicity and may cause serious health problems. This review paper focuses particularly on the recent progress and developments in this field; the working principles and basic classes of optical chemical sensors have been briefly described.

## 1. Introduction

Optical chemical sensors are devices that are used to monitor the concentration of some chemical species within a sample of concern. In recent years meaningful efforts have been made to design and develop optical sensors capable of monitoring ananalyte *in situ* with minimal disturbance to the sample matrix. The efficiency of an optical chemical sensor depends strongly on the proper choice of indicators and the sensing platforms chosen to determine the analyte. In this paper, current developments in the discipline of optical chemical sensors have been highlighted. This mini review is an effort to brief readers on the basic classes of optical chemical sensors along with different platforms and platform technologies, which are now in use for optical chemical sensing.

**Figure 1.**
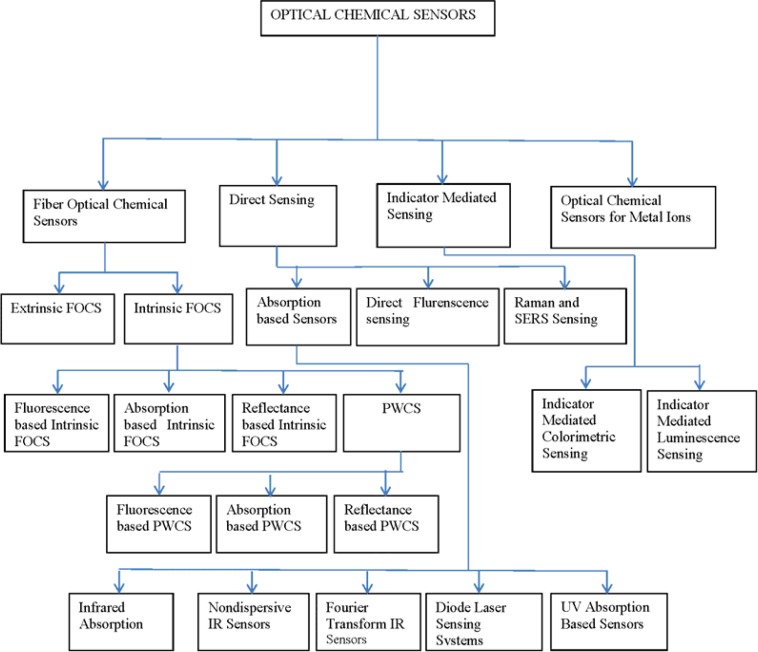
Structure of the paper.

The detection of metal ions by optical means has been a burning issue among researchers working on optical chemical sensors for a long time. In this review article, recent progress of optical chemical sensors for metal ions has also been addressed. A brief summary, mainly from the year 2009 onwards has also been given here, so that young scientists become able to get information in a single place to set their future research paths. The structure of this paper is graphically presented in Figure 1.

## 2. Fiber Optical Chemical Sensors

The initial fiber optic sensors (FOS) were designed to collect the information via fiber optics, based on the fact that alterations in a specific physical property of a medium being sensed will cause a predictable change in the light transmission characteristics of the fiber. These initial devices were capable of measuring temperature, acceleration, strain and position. In the past, the FOS technique was well known because of its main focus of physical properties, but presently people are diverting their attention to measuring chemical properties using fiber optic techniques. In this regards fiber optic chemical sensors (FOCSs) are now in operation and have been gaining escalating interest around the World.

The FOCS technique is now mushrooming at large scale owning to its numerous applications in different areas of organic, inorganic, clinical, biomedical and environmental analysis, industrial production and bioprocess control, which involves an intrinsic colour or fluorescence.

FOCS can be classified into two main groups, extrinsic and intrinsic FCOS. Extrinsic FOCS involves indirect sensing of an analyte though an optically detectible change in an immobilized indicator at the sensing end of the fiber. In this case, the fiber is not used for the sensing functions, whereas in the intrinsic case, the fiber itself plays an active role in the sensing function, during this sensing operation interaction of light with the analyte takes place with the optical fiber element. Basically, these sensors don’t measure the analyte directly but measure the effects of the analyte on some optical properties such as refractive index, absorption, emission and polarization.

FOCS sensors should have the following properties, although in practice it is difficult to design such a FOCS which has all of them [1–3]:

▪ Sensitive enough
▪ Selective
▪ Continuous and reversible or either one of these
▪ Its response to the desired analyte should be quick
▪ Stable during the course of experiment
▪ Its remote scheme should be reactive so that it may be able to sense a wide range of analytes, to avoid needing different remote-sensing schemes for each analyte

### 2.1. Extrinsic FOCS

In this sensing scheme, the analyte of concern have been determined indirectly and the fiber optic is not used for sensing purposes. Research had been conducted to determine free and total sulphur dioxide (SO_2_) in wine using FOCS [4]. In that work, a sensing membrane (4.2% Pd_2_ (dppm) 2Cl_2_, 20.8% PVC, and 75% *O*-NPOE) was deposited at the tip of a bifurcated optical fiber bundle to measure reflectance at 550 nm. The detection system of the reported sensor consisted of two cells, which hold the sample solution and an optical sensor, respectively. For the determination of SO_2_, a wine sample was mixed with sulphuric acid (H_2_SO_4_) solution in the sample cell, in which nitrogen (N_2_) was bubbled, which helps mixing of the solutions and conduction of SO_2_. For the determination of total SO_2_, a potassium hydroxide (KOH) solution was mixed with the wine in the sample cell. Linear responses up to 50 and 150 mg·L^−1^ were obtained for free and total SO_2_, with detection limits of 0.37 and 0.70 mg·L^−1^, respectively. A study was made for online detection of ammonia gas that involves meso-structured Al-MCM-41 material impregnated with bromocresol green dye using a FOCS, based on reflectance [5]. In another report, a FOCS for sensing ammonia vapours has been reported, utilizing the spectral properties of bromocresol green [6].

### 2.2. Intrinsic FOCS

As already discussed, intrinsic FOCS does not measure the analyte directly, it measures the analyte’s effects on some optical property of FOCS. Regarding the type of spectroscopy which can be used to examine a particular analyte of concern in sample solutions, intrinsic FOCS can be divided into four main categories: Fluorescence-, Absorption- and Reflectance-based intrinsic FOCS, while planar waveguide chemical sensors (PWCS) may also be considered as an individual member of the intrinsic FOCS family.

#### 2.2.1. Fluorescence-Based Intrinsic FOCS

**Figure 2.**
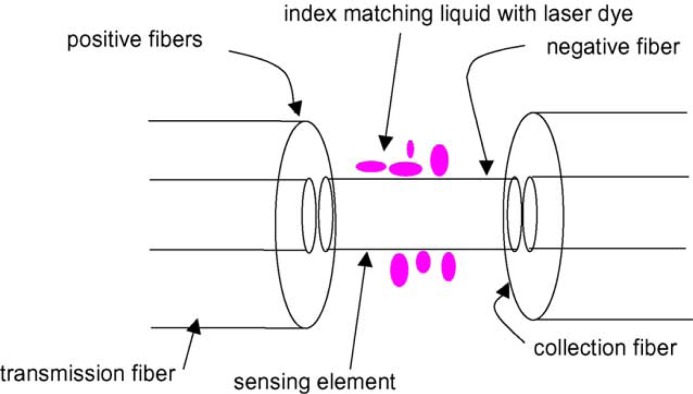
Schematic of fiber-based evanescent wave fluorescence sensor [9], reproduced by the permission of Elsevier.

To fabricate fluorescence-based FOCS, a sensor material is deposited at the distal end of the fiber or on the sensing region that is usually some declad portion of the fiber’s core. In a recent study, it was reported that plastic optical fibers are more suitable for optical chemical sensors based on the fluorescence emission of any molecule attached on the fiber surface [7]. Through the experiments, it was shown that the effect of fiber tapering was higher than the increment of length in order to increase the coupled light into the fiber core. A group of researchers conducted research to evaluate the optical properties of a fused silica fiber-optic capillary (FOCap) waveguide for fluorescence chemical sensing devices [8]. It was revealed that the FOCap shows negligible excitation light loss over a long length. A sandwiched fiber was introduced for the detection of fluorescence, emitted from a laser dye [9]. The configuration of fibers applied in the reported work is shown in Figure 2. In this work, a negative fiber was placed in between two positive fibers. One of the positive fibers was used to deliver laser light to the negative fiber. Then, the negative fibre acted as a sensing device because of the higher refractive index of glycerol then the silica core of fiber. Finally, fluorescence was guided by another positive fibre attached to the distal end of the negative fibre and detected by a CCD camera.

#### 2.2.2. Absorption-Based Intrinsic FOCS

Absorption-based FOCS may further be classified as colorimetric- and spectroscopic-based chemical sensors. These sensors involve the absorption of light by the analyte or by an indicator that causes a change in colour or produces an optically detectable signal, whose strength is directly proportional to the concentration of analyte. Colorimetric sensors respond to a change in colour of the sensing material where this change in colour has a direct relationship with the concentration of analyte. The intrinsic molecular absorption of the analyte is determined spectroscopically.

**Figure 3.**
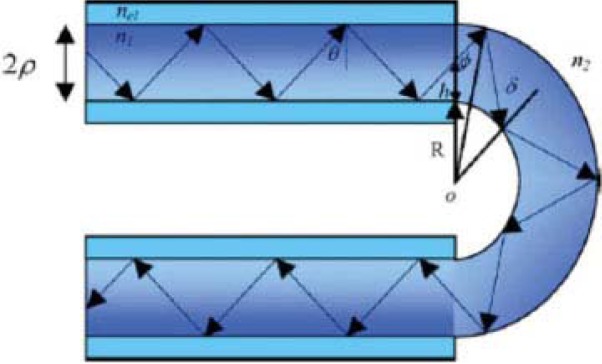
Schematic of an unclad U-bent multimode optical fiber [12]. reproduced by the permission of John Wiley and Sons.

An optical sensor based on a sensitized alumina cladding in water was designed for the detection of mercury [10]. In this work a complex of a colorimetric reagent, Ru(II)-bis-thiocyanate modulates the light intensity. In another research, a FOCS was reported to determine the low-level water content in ethanol based on evanescent field absorption spectroscopy in the infrared range of a coiled fiber optic sensor [11]. It was explained that the coiled fiber-optic sensor based on evanescent absorption spectroscopy was a feasible technology for prediction of the low level water content in bio-ethanol and other industries in both online and remote situations. However, in another strategy, a fiber optic sensor with double pass evanescent field was reported to show absorption from an unclad U-bent multimode optical fiber [12]. The schematic of the reported work is shown in Figure 3. In the work presented, one end of the fiber was polished to form an angled-tip, whereas the other end was flat and polished. Evanescent field absorption in the bending region undergoes further absorption as it reflects back from the fiber tip-air interface thus, enhancing sensitivity of the sensor. Potentially, the proposed technique may be useful for preparing the future sensing device for monitoring the various physical and chemical parameters, such as temperature, relative humidity, or chemical concentration (pH), *etc.* simply by coating suitable sensing chemicals on the surface of the sensing region of the fiber.

#### 2.2.3. Reflectance-Based Intrinsic FOCS

Reflectance-based FOCS are sensitive to the change in refractive index of optical fibers. Examples of such structures include fiber Bragg’s gratings (FBG), Fabry-Perot cavities, and metal films for surface plasmon resonance (SPR) measurements.

A hydrogen sensor based on a side-polished FBG coated with thin palladium film was made in which FBG reflectivity of 90% was fabricated in a hydrogen loaded single-mode fiber by using the phase mask writing technique of a KrFexcimer laser [13]. It was claimed that the interaction length, the diameter of the side-polished fiber and the palladium (Pd) film thickness were related to the sensor sensitivity and response time, respectively, and can be controlled independently.

A comparative study of FOCS for hydrogen based on FBG and long period gratings (LPG) coated by palladium nanolayers was conducted by a group of researchers [14]. It was concluded that both the FBG and LPG techniques can be applied for hydrogen sensing, but the sensitivity of LPG sensor was higher by about a factor 500 than that of FBG. A zeolite thin film-based fiber optic Fabry-Perot interferometric (FPI) sensor for *in situ* detection of dissolved organics in water was described in [15]. It was reported that the described sensor had different responses (time and amplitude) towards different organic molecules, with an estimated detection limit of 2 ppm for toluene, 5 ppm for 2-propanol, and 1,000 ppm for methanol.

#### 2.2.4. Planar Waveguide Chemical Sensors

In principle, in a planar waveguide chemical sensor (PWCS), a planar substrate (*i.e.*, glass, plastic or silicon) is deposited on a portion of one side of a fiber core (lengthwise). In some cases, the deposited substrate also acts as a waveguide. In some cases, an additional waveguide layer(s) is deposited onto the substrate too. Recently several configurations have been developed in which an optically detectable signal was being sensed below the planar substrate or additional waveguide layer(s). However, in some of the reported configurations the propagated light was being sensed at the sensing layer deposited on the opposite side of the waveguide layer. The comparatively robust nature of PWCS compared to its competitors, make it more practical device and design to work in harsh environments. PWCS can also be classified into three main categories: Fluorescence-based, Absorption-based and Reflectomatric PWCS.

##### Fluorescence-Based PWCS

In fluorescence-based chemical sensors, fluorescent material has been deposited on the core of fiber or on the sensing end of the FOCS and when light of a particular wavelength falls on a fluorescent molecule, it absorbs that light and emits a light with higher wavelength. In case of fluorescence-based PWCS, the fluorescent light has been detected either above or below the sensing platform. Some researchers have used waveguides to trap and carry the fluorescent light to the distal end of the fiber, where it was detected [16,17]. A team of workers also employed a generic PWCS based on fluorescence detection [16]. The proposed sensor, manufactured through both soft lithographic fabrication technique and high accuracy micro patterning technology, was successfully utilized to detect gaseous oxygen (O_2_). These techniques enable this sensor to respond more quickly and efficiently. Potentially, this sensor configuration may be adopted to develop multianalyte sensors combined with efficient fluorescence capture. Similarly, another team adopted a similar configuration to develop and characterize an integrated optical multisensor for organic pollutants in water [18].

Beside these sensors, another technique of total internal reflection fluorescence (also known as evanescent wave excitation of fluorescence) was introduced by some researchers to develop a PWCS. In this configuration, a sensing device such as prism, grating or a sensing end of the fiber was coupled with a sensing region or with the distal end of the planar waveguide [19]. At the sensing region or distal end of the planar waveguide, the evanescent field of guided light penetrates into the sensing device and is detected at the other end. This technique was more suitable for fiber optic biosensor development because of its availability for comparatively large area of evanescent field excitation. Numerous PWCSs based on fluorescence detection have been reported for different chemical and biochemical species, for example O_2_[16,17,20], polluted water [18] and carbon dioxide(CO_2_) [21,22].

##### Absorption-Based PWCS

In absorption-based PWCS platforms, a sensing layer has been deposited on the upper surface of the waveguide. Depending upon the concentration of analyte, this configuration has been based on the absorption of evanescent field intensity by the analyte or an indicator. The output intensity of the sensor lets the system estimate the concentration of analyte in the sample. In the case of colorimetric-based sensors, in response to the concentration of analyte, the sensing layer changes its colour through the evanescent field of a suitable light source. It will not be necessary to deposit a sensing layer on the waveguide if direct spectroscopy or refractometeric techniques are employed to detect the analyte of interest. In some cases transparent enrichment layers with high permeability coefficients for the analyte(s) can also be deposited [3,23].

Burke and co-researchers designed and developed a mass producible polymer waveguide chip as an enhanced platform for absorption-based PWCS [24]. In the proposed waveguide design, a refractive optic element was coupled at both ends of the waveguide, facilitating coupling to the light into and out of the waveguide. These couplers ensure that incident light falls on the sensing layer at the desired optimum angle. This was a major advantage of the described sensor configuration. Another group of researchers designed and characterized an optical sensor for gaseous ammonia based on evanescent wave absorption at room temperature with different carrier gases like argon (Ar), nitrogen (N_2_) and air [25]. In this work a dye, bromocresol purple (BCP), was deposited on fiber’s core through the sol gel process. It was reported that the described sensor showed the best response time and sensitivity when air was used as carrier gas. Numerous absorption-based PWCSs for different analytes such as gaseous ammonia (NH_3_) [25,26], O_2_[27], pH [28], water vapor [29] and multianalyte [30] were reported in recent years.

##### Refrectometric PWCS

**Figure 4.**
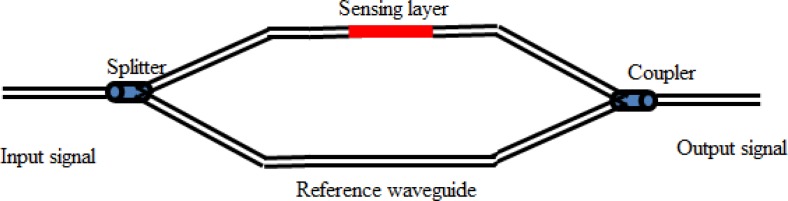
Schematic of Mach-Zehnder configration.

Refrectometric PWCS are based on a change of refractive index of a sensing layer due to the concentration of analyte in the sample. Different sensing techniques such as interferometry, surface plasmon resonance, and light coupling can be employed to introduce a refractive index change in the sensing platform. Interferometry is an optical technique that compares the differences experienced by two optical single rays, traveling along similar paths. The two most common interferometers are Mach-Zehnder and Michelson. In the Mach-Zehnder configuration, an optical signal is launched into a fiber, through a splitter input an optical signal is divided into two equal parts, both signals are launched separately into two waveguides parallel to each other. A sensing layer is deposited on one of the waveguides while other one serves as reference waveguide. When both of the waveguides are passed through a coupler, both optical signals are recombined. The schematic of Mach-Zehnder configuration is shown in Figure 4. Changes in reflective index due to the concentration of analyte cause a phase shift upon recombination of both optical signals, which is being sensed at the output end. The configuration of a Michelson interferometer is comparatively complex and may cause problems with the stability of sensor due to its high level of feedback which may arise in this arrangement. The Mach-Zehnder arrangement overcomes such problems and perhaps is the most commonly used interferometric type in fiber optical sensors [3,23,31]. In recent years, several new integrated interferometers have been proposed, developed and reported based on Mach-Zehnder arrangements for the purpose of sensing numerous chemical species in chemistry, biochemistry and the environment [32–36].

Recently, Campbell published a comprehensive overview of planar waveguide interferometers for chemical sensors [36]. In this work, he explained the principles of operation of waveguide and different types of interferometers used for chemical sensing. Meanwhile, another reviewer reported on the progress in the design and development of Mach-Zehnder interferometric platforms [34]. He also explained some strategies that proposed and employed by researchers to improve the sensitivity of sensors. Another research group proposed a configuration for a Mach-Zehnder interferometer entirely composed of liquid core waveguides [37]. The proposed configuration was employed to develop an opto fluidic asymmetric Mach-Zehnder interferometer based on anti resonant reflecting optical waveguides with a liquid core. The experimental results demonstrated that interferometers with good visibility have the potential to achieve the theoretical results.

In recent years, various integrated optical chemical and biochemical sensor schemes based on Young interferometric [38–46], Michelson interferometeric [47–49], and other interferometic techniques [50–53] have been proposed. Basically, Young’s interferometer sensors are based on either slab or strip waveguides. There are two types of Young interferometer sensors based on slab configurations. One of them consists of two planar core layers separated by a low index buffer layer while the other one consists of double slits to yield two optical signals that are simultaneously coupled through an integrated grating into a signal planar waveguide for sensing and referencing purposes. Stripe-based Young interferometer sensors contain a sensing and referencing arm connected through a coupler [38–41,44].

Recently, a group of researchers characterized the performance of a Young’s interferometer sensor through a prism chamber assembly which was designed and developed by them. It was reported that the fringe contrast depended on the ratio of the slit width to the spatial periodicity of the sensor; the regular high contrast interference pattern can be readily detected as the slit width is smaller than the spatial periodicity. Based on a brief ringent silica rib waveguide, an integrated optical refractometric sensor has been introduced recently [54]. In the reported sensor, to get rid of undesired absorptions, a uniform high index titanium dioxide (TiO_2_) film was deposited on the whole waveguide except the sensing region located in the center. Through experiments, it was observed that the presence of the analyte affects the refractive index which was monitored through the polarimetric interference scheme by observing the accumulated phase difference between transverse electric and transverse magnetic mode.

## 3. Direct Sensing

The continuous monitoring of an analyte (particularly, for gas species), through optical means, can be categorized into two subcategories, named as direct sensing schemea and reagent-mediated sensing scheme. In direct sensing scheme, some intrinsic optical property (such as absorption, fluorescence, *etc.*) of an analyte is monitored for sensing purposes. When the analyte does not have an adequate intrinsic optical property which may be monitored directly, the reagent-mediated sensing scheme is applied. In this section, recent progress in the field of optical chemical sensors based on direct sensing schemes is reported. The optical spectroscopic techniques and operational principles are also discussed here. Based on the currently employed spectroscopic techniques to design and fabricate an optical chemical sensor based on direct sensing schemes, this section is further divided into three subsections, namely direct absorption-, fluorescence-, and Raman-based sensing. The next section we will address the recent progress in the field of optical chemical sensors based on reagent-mediated sensing schemes.

### 3.1. Absorption-Based Sensor

In case of absorption based sensors, radiation wavelength ranges from 200 nm to 4 μm can be utilized for sensing purposes. This section covers direct absorption-based optical chemical sensors.

#### 3.1.1. Infrared Absorption

Infrared (IR) absorption spectroscopy has been a widely adopted technique. In recent years, IR spectroscopy has been exploited for the purpose of sensing many gases such as chlorine (Cl_2_), methane (CH_4_), carbon dioxide (CO_2_), carbon monoxide (CO), nitric oxide (NO) and nitrogen dioxide (NO_2_). The absorption-based optical chemical sensor consists of infrared sources, special types of fibers which have zirconium fluoride or silver halide core that are capable to transmit IR singles to acquire IR spectra via optical fibers and an optical filter to select a specific absorption wavelength and optical detector. Infrared absorption spectroscopy obeys the Lambert-Beer law. As compared to visible (VIS) absorption, the molecular absorption is very low, so a large “cell length” is also required to enhance the sensitivity of the sensor. IR absorption spectroscopy covers the ranges from IR (from 800 nm to about 2,500 nm) to near infrared (NIR; from 2.5 to 20 μm) to detect the concentration of molecular trace gases. There are two types of direct optical chemical sensing methods which are nondispersive and dispersive methods. In nondispersive method, a single laser line is used to gain analytical information. This method is only applicable when the molecules of analyte have a unique absorption band and a proper laser line. While in dispersive method, a whole spectrum is acquired and deconvoluted, mostly by the Fourier transform technique [3,31,55].

#### 3.1.2. Nondispersive IR Sensors

In principle, in nondispersive IR sensors, a band filter is used to select the specific analyte’s absorption wavelength. The nondispersive IR gas analyser is employed to determine the increasing concentration of CO_2_ in seawater to assess the effects of increasing CO_2_ in the global carbon circle [56,57]. A method was proposed to measure total dissolved inorganic carbon precisely and accurately in seawater [57]. The proposed method was based on continuous gas extraction of acidified seawater, which was pumped through an extraction chamber at a constant flow rate. In this model, a nondispersive IR gas analyser was employed to determine the purged carbon dioxide. It was reported that the proposed method had precision of 0.05% along with accuracy of about 0.1%. Both precision and accuracy of the reported method are comparable to the standard coulometric technique, and its response time is three times faster than the standard coulometric technique. Another system was developed to monitor the concentration of total dissolved carbon dioxide in continuously flowing seawater streams through a nondispersive IR analyser [58]. The reported system continuously acidifies the sample stream and quantitatively strips it to evolve carbon dioxide out into the membrane container. The carbon dioxide in the strip gas stream was then analyzed using a nondispersive IR gas analyzer. It was reported that the reported system has accuracy and precision batter than ±0.1% with a response time of 6 s.

#### 3.1.3. Fourier Transform IR Sensors (FTIR)

The FTIR technique can be used to obtain an emission infrared spectrum from a sample, and it can also be employed to convert the raw data into an actual spectrum. A FTIR spectrometer consists of an interferometer which is used to generate an interferogram from IR emission, and then it is employed to obtain the spectrum. The capability of a FTIR spectrometer to collect spectral data over a wide spectral range simultaneously and then simultaneously process it, make it superior to its competitors. Another advantage of FTIR spectroscopy is it depends upon the characteristic absorbance of certain molecular vibrations in the sample. Therefore, FTIR imaging does not require any additional dye or labeling methods for visualization of different chemical components in the sample [3,59,60]. Recently, a FTIR imaging technique has been employed to analyse oxygen inhibition in photopolymerizations of hydrogel micropatterns [61]. Through experiments, it was observed that oxygen inhibition during polymerization was reduced by increasing the amount of photoinitiatoror increasing UV intensity. A microreactor-FTIR imaging system was developed to obtain *in situ* transmission FTIR analyses of working catalysts with both spatial and temporal resolution [62]. In that work Micro-Electro-Mechanical Systems and microfabrication technologies were used to design and fabricate a microreactor with geometric and optical properties ideal for coupling with a high-throughput transmission FPA-FTIR system. CO adsorption and oxidation on Pt/SiO_2_ were used as a model catalyst system. A 128 × 128 pixels MCT focal plane array was used in conjunction with a Nicolet Magna-860 FTIR spectrometer. The optical setup provided an analysis dimension of about 400 × 400 mm for each pixel. The FPA was driven at a frame capture rate of 1,600 Hz while running the FTIR at a mirror velocity of 0.3165 cm/s and a resolution of 4 cm^−1^. The trigger from the FTIR was first passed through a custom built trigger box which delays the periodic transistor-transistor logic signal by a factor of 8. This forced delay allows the FPA time to transfer the data for each captured spectrum to the computer. With these parameters, 2,484 frames were required to describe a full spectrum over a range up to 2,527 cm^−1^. Raw data from the FPA were captured using WinIR 3.7.0.0. Data were processed post capture with the combination of another image manipulation software program for transforming and averaging, as well as in-house written software to extract and process the data. Each FPA spectrum here was averaged over 6 scans and corrected using a triangular apodization. It is reported that propagation of adsorbed species down the length of the microreactor and fractional coverage were quantified during pulsed chemisorptions experiments.

#### 3.1.4. Diode Laser Sensing Systems

**Figure 5.**
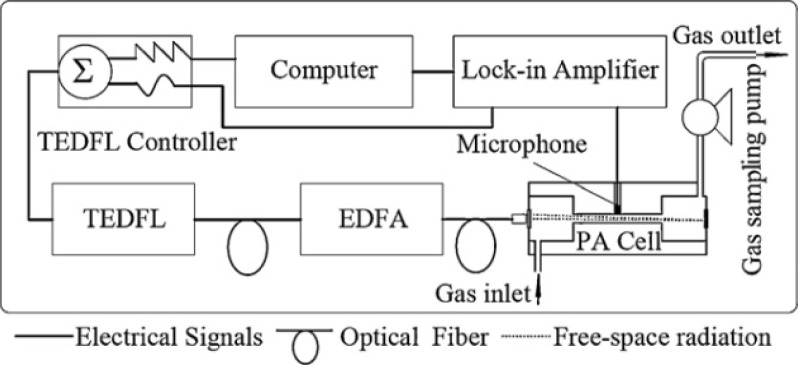
The schematic of photoacoustic spectrometer [64], reproduced by the permission of Elsevier.

In last few decades, increasing man-made pollution in the Earth’s atmosphere have brought about considerable interest in the design and development of a monitoring system capable of monitoring trace gases more precisely and accurately in real time to monitor impurities, especially for the trace gases in Earth’s atmosphere. Moreover, there is the demand of time to detect and quantify trace gases in different processes such as chemical, biochemical, clinical, agricultural, and industrial processes in real time. It is not to say that FTIR and other optical analytical spectroscopic techniques are not capable of precise *in situ* monitoring, but laser spectroscopy is one of the choices to monitor trace gases due to its high sensitivity and specificity. However, the laser source characteristics such as available wavelengths, tunability, linewidth, power, operation temperature, *etc.*, as well as the combinations with appropriate sensitive detection schemes are crucial for the success of laser-based sensing [63]. Recently, a multi-gas photoacoustic spectrometer based on the tunable fiber laser was developed for simultaneous and continuous monitoring of H_2_O, ethyne (C_2_H_2_), CO and CO_2_ in gas mixtures [64]. In the described work, a near-IR tunable erbium-doped fiber laser was used as a light source. A 980 nm pump diode laser was adopted and a 7 m erbium-doped fiber was used as a gain medium. To ensure the laser propagation is unidirectional in the fiber laser loop an optical isolator was employed. The laser operation wavelength can be tuned by adjusting the voltage load on the Fabry-Perot tunable filter (FFP-TF) in the laser loop. To ensure that erbium-doped fiber amplifier is fully saturated, the near-IR tunable erbium-doped fiber laser was operated in continuous wave output power mode. The output of near-IR tunable erbium-doped fiber laser was divided into two branches through a 1 × 2 fiber splitter. 1% of the optical power entered a hydrogen cyanide gas reference cell, which was used to calibrate the laser wavelength to the selected multi-target gases absorption lines, while the remaining 99% of the laser power was amplified by computer-controlled erbium-doper fiber amplifier. The operating wavelength of near-IR tunable erbium-doped fiber laser was scanned continuously with different scan rate ranges from 1,520 nm to 1,610 nm. Wavelength modulated light from the near-IR tunable erbium-doped fiber laser was amplified by erbium-doper fiber amplifier and coupled into the photoacoustic cell with a double-pass configuration through a fiber collimator. The gas sampling pump controlled the targeted gas entering the photoacoustic cell. The photoacoustic signal was detected by a microphone placed in the middle of the photoacoustic cell resonator. The photoacoustic signal was fed through the lock-in amplifier and then transmitted to the computer where a computer program controlled the acquired and processed data. A schematic of the photoacoustic spectrometer is shown in Figure 5. Through this work, a minimum detection limit of 70 ppm for H_2_O, 2 ppb for C_2_H_2_, 4 ppm for each of CO and CO_2_ was observed at atmospheric pressure. Based on temperature tuning, diode laser absorption spectroscopy has been employed for the detection of CO and CO_2_in vehicle emissions in another reported work [65].

In recent years, numerous research projects have been conducted to develop a laser diode system based on a quantum cascade laser (QCL) to monitor trace gases or other chemical species at room temperature. Besides ultra-sensitivity and highly selectivity, the capability to operate QCL over a wide range of wavelengths (from ∼3 μm to ∼20 μm) at room temperature makes it unique among its competitors [66,67]. A cavity ring-down spectroscopy (CRDS)-based NO sensor utilizing a mid-IR pulsed QCL oscillating near the 5.26 μm wavelength has been reported [68]. In that work, an effective optical path length of 2.1 km was achieved in a 50 cm long cell using high reflectivity mirrors. In combination of a particle filter and a membrane gas dryer, stable and sensitive measurement of NO in exhaust gas was achieved for more than 30 min with a time resolution of 1 s. From the results of this study, it was concluded that a laser based NO sensor can be used to measure NO in exhaust gas over a dynamic range of three orders of magnitude. Research has been conducted to investigate the simultaneous use of two QCL for on-line detection in high performance liquid chromatography [69]. Through this research, it was reported that all investigated species could be chromatographically separated, detected and quantified in complex matrices. These analyses were conducted over seven different wine and grape juice samples. It was reported that QCL based systems offer a significant advantages for on-line monitoring over FTIR spectrometers.

#### 3.1.5. UV Absorption-Based Sensors

Diode laser sensing spectroscopies are employed to monitor trace gases in the Earth’s atmosphere, while UV absorption spectroscopy can mainly be employed to detect and quantify pollutant elements such as metals, hydrocarbons and volatile organic compounds in the Earth’s atmosphere [23]. Fiber optic sensors based on ultraviolet absorption to monitoron-board automobile hazardous exhaust emissions are reported [70–72]. The light loss of UV/VIS spectrumhas been utilized through a gas cell to determine the level of absorption for three exhaust gases (*i.e.*, NO_2_, SO_2_, NO) by a group of researchers [72]. Through this research, theoretical results were verified. The response time of the reported sensor was less than 4 s. This sensor was operated over a wide range of concentrations and a detection limit was in the order of 1ppm for both NO_2_ and SO_2_ and 26ppm for NO.

### 3.2. Direct Fluorescence Sensing

Direct fluorescence sensing is mainly employed in biomedical applications. Auto fluorescence spectroscopy is a widely employed technique for noninvasive scanning of precancerous development in the epithelium where most human cancers originate. In principle, epithelial fluorescence is mainly determined by the emission of the intrinsic fluorophores reduced nicotinamide adenine dinucleotide (NADH) and flavin adenine dinucleotide (FAD). The fluorescence of the both of these species can be used to monitor cell activity. For example, the ratio of free and bound NADH, which is a good indicator of the cells’ metabolic state, can be differentiated by their different lifetimes [23,73,74]. Research has been conducted to measure the free-bound NADH fluorescence in epithelial cells [74]. It was reported that time-resolved fluorescence measurements provide an important contrast mechanism for detecting epithelial precancer. The cellular redox ratio, NADH and FAD lifetime, and subcellular morphology imaging in three dimensions are combined to diagnose intrinsic sources of metabolic and structural contrast *in vivo* at the earliest stages of cancer development [75]. It was reported that the redox ratio significantly decreased in the less differentiated basal epithelial cells compared to more mature cells in the superficial layer of the normal stratified squamous epithelium. However, the redox ratio was not significantly different between the superficial and basal cells in precancerous tissues. Recently, a compact point-detection fluorescence spectroscopy system has been developed to quantify the intrinsic fluorescence redox ratio in an *ex vivo* orthotopic brain tumor rat model [76]. In the reported system the measured fluorescence spectra was processed by two methods. First spectra wasanalyzed using a spectral filtering modulation method to derive the intrinsic fluorescence redox ratio and then a multivariate method was used to statistically classify normal tissues and tumors. It was concluded that although the first method offers quantitative tissue metabolism information, these condmethod provides overall diagnostic accuracy. Both methods possess complementary capabilities for understanding cancer development and noninvasively delineating brain tumor margins.

In recent years, fluorescence spectroscopy has also been applied for environmental monitoring purposes [77–79]. Fluorescence excitation emission matrix spectroscopy has been employed to determine pH, conductivity and dissolved organic carbon in river water [77]. In another work, lifetime *versus* emission wavelength plots were utilized for *in situ* monitoring of different oil populations due to petroleum-bearing fluids [79].

### 3.3. Raman and SERS Sensing

Raman spectroscopy is an emission technique based on inelastic scattering of absorbed light. When incident radiation from a monochromatic source falls on a sample, some of the radiation is absorbed by the sample and some of the radiation is scattered. There are two types of scattered radiations, scattered radiation along with some more radiation with the same frequency as that of incident radiation known as elastically scattered radiations or Rayleigh radiations, while some of the scattered radiations with different frequency from that of incident radiation is also observed, which is known as inelastically scattered radiation or Raman radiation. Like spectra obtained from other vibrational techniques, Raman spectra can be treated as a compound's fingerprint which makes Raman spectroscopy a superior technique for the identification of many compounds [80,81]. In recent years, Raman spectroscopy has been employed for fast detection and identification of counterfeit medicines [82–84]. One team of researchers experimentally demonstrated that Raman spectroscopy, in combination with chemometry, was notonly able to discriminate between genuine and counterfeit tablets but also produced a “chemical fingerprint” of different types of counterfeit medicines, which is helpful in determining the relationships between different samples [82]. It was suggested that a combination of Raman spectroscopy and multivariate clustering can assist in the forensic investigation of the sources and trade routes of counterfeit medicines. Raman spectroscopy is also a useful technique for clinical applications. Recently, Raman spectroscopy has been applied to diagnose different breast diseases, including breast cancer [85,86]. A study has been conducted to examine normal and malignant human breast tissues by employing Raman spectroscopy [87]. The results obtained through this study demonstrated a sensitivity of 83%, a specificity of 93%, a positive predictive value of 36%, and a negative predictive value of 99% for distinguishing cancerous from normal and malignant tissues.

The low scattering cross-sections of Raman spectroscopy can be improved to a large extent (up to 15 orders of magnitude in comparison to the “normal” Raman scattering) by using Surface Enhanced Raman Scattering (SERS). In principle, an increase in Raman spectroscopy intensity due to the SERS causes an enhancement ofthe electric field on the metal surface. The surface-enhanced effect is strongest for gold or silver nanostructures, because of the interaction of the localized surface plasmon generated on the metal surfacewith the vibrational levels of the molecule. There aretwo principal conditions required for SERS to be observed: the presence of a suitable SERS active nanostructured metal surface and the sample under investigation must be immobilized or in close proximity to the surface. Recently, due to the advances in materials fabrication and better understanding of the details of the plasmonic interaction, SERS use is increasing in diverse fields such as biomedicine and environmental analysis [23,80,88–92].

## 4. Indicator-Mediated Sensing

In the situations when an analyte does not have enough spectroscopic optical response to monitor it directly in the sample, sensing can be accomplished by introducing a suitable indicator into the sensing system whose spectral properties reflect the analyte’s concentration. Practically all known in dicator-mediated sensors are based either on absorption or on luminescence measurements. In recent years, indicator-mediated sensing was mainly based on solid-phase immobilization matrices where the reagent dye can be adsorbed, covalently orionically attached, or simply encapsulated in a solid matrix that is permeable for the analyte [3,23].

### 4.1. Indicator-Mediated Colorimetric Sensing

The indicator-mediated colorimetric sensing scheme can be employed when the analyte has not adequate spectroscopic properties to monitor it directly. The stated scheme has been employed successfully to determine many chemical properties and species, including pH and CO_2_. As most of the reported optical colorimetric chemical pH sensors are operated in a narrow pH range, researchers from all over the World are trying to improve the operational pH range using several pH indicators with different acid-base equilibrium (pKa) constants. A broad range optical pH sensor based on multiple pH indicators has been reported [93]. In that work, the pH requirements to design multi-indicator-based optical pH sensors has also been addressed, including ΔpKa between the indicators and the concentrations of the indicators. Recently, a team of researchers developed a ratiometric pH nanosensor with tuneable pKa [94]. In this work, two pH-sensitive fluorophores (fluorescein isothiocyanate dextran (FITC-D) and Oregon Green dextran (OG-D)) and a reference fluorophore (5-(and-6)-carboxy tetramethyl rhodamine dextran (TAMRA-D)) were entrapped in a biocompatible polymer matrix to determine pH over an extended dynamic range of pH from 4.0 to 7.5. In another work, a benzophenoneazo dye was used to monitor pH over the range from 7.9 to 9.3 [95].

Indicator-mediated colorimetric sensing schemes may also be employed to determine the concentration of CO_2_ by monitoring pKa in those chemical reactions in which the value of pH depends upon the concentration of carbonic acid (H_2_CO_3_) generated by CO_2_. Recently, a polymer-based sensing layer to monitor CO_2_ was reported [96]. The sensing layer was based on the pKa of phenol and its derivative *p*-nitro-phenol. It was reported that in the presence of carbon dioxide, both phenol and its derivative *p*-nitro-phenol were sensitive to protonation, which results in changes in absorption and refractive index. Some other researchers used α-naphtholphthalein, thymol blue and cresol red for CO_2_ sensing [97–99]. A pH indicator has also been used to monitor the change in basicity due to the reactions between nonvolatile primary amines with formaldehyde [100].

### 4.2. Indicator-Mediated Luminescence Sensing

Luminescence can be observed when the energy by an electronically excited state species is released in the form of light. Depending upon whether the excited state is singlet or triplet, the emission is called fluorescence or phosphorescence. Both fluorescence and phosphorescence are at least tri-parametric (*i.e.*, excitation wavelength, emission wavelength and emission intensity). Intrinsically luminescence is more sensitive than absorption as a sensing technique for many applications, luminescence based sensors offer higher sensitivity than absorption-based sensors [3]. Like absorption-based sensors, luminescence-based sensors for pH, CO_2_, and ammonia are also based on the changes in luminescence of a pH indicator. Recently, a pH sensing device based on indicator-mediated luminescence sensing scheme was reported [101]. In that work, a fluoroionophore substituted with a silane coupling agent (KBH-01-Si) was successfully synthesized. A mesoporous silica thin film was fabricated by the evaporation-induced-self-assembly (EISA) process. To fabricate a pH sensor, KBH-01-Si was attached with the mesoporous silica thin film by covalent bonding via a sol-gel grafting method. Potentially, the reported sensor may be used in biological and medical diagnosis. A team of researchers utilized a fluorescent pH indicator dye (8-hydroxy-1,3,6-pyrenetrisulfonic acid trisodium salt, HPTS, PTS^−^), tetraoctylammoniumcation (TOA^+^), and tetraoctylammonium hydroxide (TOAOH) immobilized within an ethyl cellulose membrane to examine transport-reaction processes controlling CO_2_ on the seafloor [102]. It was reported that a novel TFP-TriMOS-based xerogel was utilized to fabricate a fluorinated sensing film to determine dissolved oxygen [103]. In the reported work, dissolved oxygen was sensed by its quenching effect on the fluorescence of Ru(bpy)_3_^2+^ immobilized in the sensing film. TFP-TriMOS caused an enhancement of the surface hydrophobicity and oxygen permeability of the fluorinated sensing film because the majority of surface Si-OH groups were replaced by Si-CH_2_CH_2_CF_3_ groups, which may effectively impart oxygen from dissolved water and hence improve the sensor performances. Ru(bpy)_3_^2+^-doped hybrid fluorinated organically modified silicates (ORMOSILs), Pt(II) porphyrin dye immobilized on surface layer protein matrices and Pt(II) meso-tetrakis(pentafluorophenyl)porphyrin (PtTFPP) embedded in an *n*-octyltriethoxysilane (octyl-triEOS)/tetraethylorthosilane (TEOS) composite xerogel have also been used to develop OCS for oxygen [104–106].

## 5. Optical Chemical Sensors for Metal Ions

In the last few decades, researchers from all over the World have dedicated their efforts to fabricate optical chemical sensors to determine metal ions not only in the laboratory but also in real samples. This is due to the fact that optical chemical sensors potentially have the ability to analyse the sample *in situ* and in real time with minimal or no disturbance to the sample. The immunity to electricity of the optical chemical sensing system also makes them safer and more suitable for work in harsh environments. However, some disadvantages also exist. Some of them have smaller dynamic ranges and low analytical selectivity. Moreover, there are only few optical indicators available for metal ions or other analytes of concern, which may minimize the number of analytes that can be measured by optical chemical sensors. In this section, recent progress (from 2009 to date) in the field of optical chemical sensors for metal ions has been addressed. Irrespective to sensing scheme employed to determine the analyte, all data have been summarized in a tabular form. To the best of our knowledge, all important parameters including: (a) analyte of concern, (b) reagents and indicators, (c) immobilization material (if any), (d) sample or solution in which analyte was analysed, (e) linear response range, (f) limit of detection, (g) working pH value or range in which sensor’s performance was optimized and (h) detection method have been listed here in a single citation for the first time.

Due to its stability and high toxicity, the determination of lead in the environment is attracting the attention of researchers. In Table 1, priority has been given to those optical chemical sensors which have been reported to analyse lead. It can be seen that some of the sensors are selective towards lead in the presence of other metal ions, but still more sensitive and selective sensors are needed.

Copper is another important heavy metal ion, which has always been under consideration. Literature survey highlight that although in the recent years many efforts have been reported to trace out copper in the sample but still deep rooted efforts are required to design simultaneously selective and sensitive sensor for the detection of copper.

Detection of mercuric ions in the environment has always been challenging because of its liquid nature. In the presence of other metal ions, its determination is very difficult. Globally, researchers are to produce a highly selective optical chemical sensor for mercuric ion without compromising the sensitivity. Sensors for zinc, silver, cadmium, iron, nickel and other metal ions are also listed afterward. Seeing this table it is very easy to understand that still sensitivity, selectivity, linear response rang and pH ranges of the optical chemical sensors for the detection of a particular metal ion are the big questions for today researchers.

## 5. Summary and Future Trends

In this review article, recent progress in the field of optical chemical sensors has been summarized. Basic classes and sensing techniques which are now in use for optical chemical sensing have also been addressed in detail. It is clear from the literature cited here that remarkable efforts had been done to determine analytes *in situ* and in real time with minimal or no disturbance of the sample of concern. It is obvious that the basic sensing principles of optical chemical sensors are mature enough and in coming years major changes in sensing mechanisms are not expected. Researchers from all over the World are trying to improve the design and sensing schemes of optical chemical sensors. It is the fruit of these efforts that now a large number of more sensitive optical chemical devices, including fiber-optic capillary waveguides, microfluidics and lab-on-a-chip devices are available and in the near future more and smart sensitive devices will be accessible. Researchers are also focusing on producing fast responding, sensitive, selective, and portable optical chemical sensors for continuous monitoring of the samples. It is hypothetical that in the future some portable optical chemical devices will be available to analyse a sample on site. Recent advances in material engineering are attracting the attention of researchers to develop an indicator or reagent which may be used for multi-analyte determination in a single sample, so that, the dream to determine the multi-analytes in the sample through an optical chemical sensor become reality.

## Figures and Tables

**Table 1.** Recently reported optical chemical sensors for metal ions.

	**Analyte**	**Reagent or Indicator**	**Immobilization material**	**Sample**	**linear response range**	**Limit of detection**	**pH**	**Selectivity**	**Detection method**	**Ref.**
1	Pb(II)	Ionophore (lead IV), proton-selective chromoionophore (ETH 5294) and lipophilic anionic sites (KTpClPB)	PVC membrane	Water sample	1.26 × 10^−8^ to 3.16 × 10^−5^ mol·L^−1^	8.97 × 10^−9^ mol·L^−1^	7.0	Na(I), K(I), Mg(II), Cd(II), Hg(II), Ag(I)	Absorption	[107]
2	Pb(II)	Triazolothiadiazine	PVC membrane	Water sample	5.0 × 10^−8^ to 3.8 × 10^−4^ M	2.2 × 10^−8^ M	5.0–6.0	Good selectivity for Pb(II) over other metal ions	Fluorescence	[108]
3	Pb(II)	2,7-bis(2-Arsenophenylazo)-1,8-dihydroxynaphthalene-3,6-disulphonic acid (arsenazoIII or ASA III)	XAD-16	Aqueous solution	0.2 to 20.7 ppm	0.01 ppm	5.0	Non-selective	Reflectance	[109]
4	Pb(II)	Synthesized 5,10,15,20-tetra-(3-bromo-4-hydroxyphenyl)porphyrin (TBHPP)	PVC flim	Aqueous solution	5 × 10^−6^ to 4 × 10^−4^ mol·L^−1^	4 × 10^−8^ μM	6.0–7.8	Selective for Pb(II) over Na(I), K(I), Ca(I), Mg(II),	Fluorescence	[110]
5	Pb(II)	4-Hydroxysalophen	--	Aqueous solution	1.0 × 10^−3^ to 1.0 × 10^−7^ mol·L^−1^	8.6 × 10^−8^ mol·L^−1^	3.7	Excellent selectivity toward Pb(II) w.r.t other metal ions	Absorption	[111]
6	Cu(II)	Functional CdS nanoparticles	--	Water sample	0.09 to 27.0 μg·L^−1^	3.2 ng·L^−1^	4.9	Selectivity toward Cu(II) w.r.t other metal ions expect the Fe(III)	Fluorescence	[112]
7	Cu(II)	4,5-Disubstituted-1,8-naphthalimide derivatives	--	Aqueous solution	--	2.48 ppm	4.9–13	Selectivive toward Cu(II) w.r.t other metal ions	Fluorescence	[113]
8	Cu(II)	4,5-Disubstituted-1,8-naphthalimide derivatives	--	Aqueous solutions	--	0.52 ppm	4.2–13	Selectivive toward Cu(II) w.r.t other metal ions	Absorption	[113]
9	Cu(II)	Functionalized-8-hydroxyquinoline	--	Water samples	--	4.7 nM	7.2	Selective for Cu(II)over other ions	Fluorescence/Absorption	[114]
10	Cu(II)	poly(2,5-di(Propyloxysulfonate)-1,4-phenyleneethynyl)-ene-9,10-anthrylene	--	Buffer solution	0.0 to 100,000 μmol·L^−1^	5.0 nmol·L^−1^	7.5	Sensitive but less selective	Fluorescence	[115]
11	Cu(II)	1-Phenyl-1,2-propanedione-2-oxime thiosemi-carbazone (PPDOT)	Triacetylcellulosemembrane	Water sample	7.5 × 10^−6^ to 2.0 × 10^−4^	8.0 × 10^−7^ μM	5.8	Sensitive but less selective	Absorption	[116]
12	Cu(II)	4-[(*E*)-2-(4′-Methyl-2,2′-bipyridin-4-yl)vinyl]phenol	Sol-gel matrix	Spring water samples	2.5 to 50 μmol·L^−1^	4.7 × 10^−7^ μM	5.0	Poor selectivity	Fluorescence	[117]
13	Hg(II)	tris[2-(4-Phenyldiazenyl)phenylaminoethoxy]cyclotriveratrylene (TPPECTV)	Triacetylcellulose membrane	River water	0 to 2.0 × 10^−5^ M	5.0 μM	5.8	Less selective	Absorbance	[118]
14	Hg(II)	tris[2-(4-Phenyldiazenyl)phenylamino)ethoxy]cyclotriveratrylene (TPPECTV)	PVC film	River water	1.0 × 10^−6^ to 2.5 × 10^−4^ M	0.5 μM	7.0	Selective for Hg(II) inpresence of other ions	Absorbance	[119]
15	Hg(II)	4-Ethyl-5-hydroxy-5,6-dipyridin-2-yl-4,5-dihydro-2H-[1,2,4]-triazine-3-thione	PVC membrane	Tap water	5.0 × 10^−10^ to 5.0 × 10^−5^ M	1.8 × 10^−7^ μM	5.5	Highly selective for Hg(II)	Fluorescence	[120]
16	Hg(II)	Quinolin-8-ol-*p*-[10′,15′,20′-triphenyl-5′-porphyrinyl]-benzoate	--	Aqueous ethanol	3 × 10^−7^ to 2 × 10^−5^ M	2.2 × 10^−8^ μM	5.0–9.0	Excellentselectivity to Hg(II)over transitionmetalcations except Cu(II)	Fluorescence	[121]
17	Hg(II)	2,6-Pyridinedicarbox-aldehydebis(*o*-hydroxyphenylimine)	--	Aqueous solutions	5.0 × 10^−5^ to 2.5 × 10^−8^ M	5.0 × 10^−8^ μM	6.5–7.5	Highly selective for Hg(II) over Na(I), Ca(I), Mg(II) and Fe(II)	Luminescence	[122]
18	Hg(II)	(1*Z*,2*Z*)-N′1,N′2-dihydroxy-N1,N2-dipyridin-2-yl-ethanediimidamide	Agarose membrane	Aqueous solutions	5.78 × 10^−9^ to 1.05 × 10^−3^ M	1.71 × 10^−9^ μM	4.0	Highly selective to Hg(II)ion	Absorption	[123]
19	Hg(II)	2-[(2-sulfanylphenyl)ethanimidoyl]phenol	Agarosemembrane	Water sample	1 × 10^−2^ to 1 × 10^−5^ mol·L^−1^	1 × 10^−6^ mol·L^−1^	4.5	Presence of other has minor effects on the selective for Hg(II)	Absorption	[124]
20	Hg(II)	4-Phenyl-2,6-bis(2,3,5,6-tetrahydrobenzo[b][1,4,7]-trioxononin-9-yl)-pyrylium perchlorate	Sol-gel layer	Aqueous solution	1.52 × 10^−9^to 1.70 × 10^−2^ M	1.11 × 10^−9^ μM	5.0	Selective for Hg(II) in other cations	Absorption	[125]
21	Hg(II)	4-Phenyl-2,6-bis(2,3,5,6-tetrahydrobenzo[b][1,4,7]trioxo-nonyn-9-yl)pyrylium perchlorate	PVC membrane	Aqueous solution	2.95 × 10^−10^ to 3.20 × 10^−3^ M	1.01×10^−1^° μM	5.0	Selective for Hg(II)ions over other metal ions	Absorption	[126]
22	Hg(II)	Rhodamine B derivative (RND)	PVC membrane	Environmental water	1.0 × 10^−9^ to 2.0 × 10^−3^ M	8.1 × 10^−10^ μM	6.5	Selective for Hg(II)ions with respect to other cations	Fluorescence	[127]
23	Hg(II)	4-[(*E*)-2-(4′-methyl-2,2′-bipyridin-4-yl)vinyl]phenol	Sol-gel matrix	Spring water samples	2.5 to 50 μmol·L^−1^	2.9 × 10^−7^	5.0	Poor selectivity	Fluorescence	[117]
24	Hg(II)	Tetrathia-12-crown-4 (TT12C4)	PVC membrane	aqueous solution	9.5 × 10^−9^ to 1.8 × 10^−5^ mol·L^−1^	8.1 × 10^−10^ mol·L^−1^	7.0	Selective and fully reversible	Absorption	[128]
25	Hg(II)	4-(2-Pyridylazo)-resorcinol (PAR)	tri-(2-Ethylhexyl) phosphate plasticized cellulose triacetate matrix	aqueous solution	0.22 to 1.32 μg/mL	0.11 μgm^−1^·L^−1^	7.5	Sensitive but non-selective	Absorption	[129]
26	Hg(II)	Hexathiacyclooctadecane	PVC membrane	Water samples	2.1 × 10^−7^ to 1.2 × 10^−4^ mol·L^−1^	2.0 × 10^−7^ mol·L^−1^	4.0	Selective and disposable	Absorption	[130]
27	Cd(II)	2-Amino-cyclopentene-1-dithiocarboxylic acid	Triacetylcellulose membrane	Water samples	3.0 × 10^−6^ to 3.4 × 10^−4^ M	1.0 × 10^−6^ M	6.2	Selective at certain pH value	Absorption	[131]
28	Cd(II)	4-Hydroxysalophen	Triacetyl cellulose	Aqueous solution	1.0 × 10^−6^ to 5.0 × 10^−2^ M	5.3 × 10^−7^ mol·L^−1^	6.0	More selective for Cd(II) than Nafion sensor	Absorption	[132]
29	Cd(II)	1-(2-Pyridylazo)-2-naphthol (PAN)	Tri-(2-Ethylhexyl) Phosphate plasticized Cellulose Triacetate matrix	Water samples	250 ng·mL^−1^ to 5,000 ng·mL^−1^	250 ng·L^−1^	7.5	Selective at certain pH value	Absorption	[133]
30	Cd(II)	4-Hydroxysalophen	PVC membrane	Water samples	1.0 × 10^−6^ to 1.0 × 10^−1^ mol·L^−1^	8.4 × 10^−7^ mol·L^−1^	2.8–8,1	Sensitive and less selective	Absorption	[134]
31	Cd(II)	Rhod-5N	--	Buffered solutions	--	0.45 μg·L^−1^	7.0	Good selectivity towards Cd (II) in the presence of Pb(II)	Fluorescence	[135]
32	Ag(I)	3,3′,5,5′-Tetramethylbenzidine (TMB)	--	Buffered solutions	--	50 nM	4.0	Selective toward Ag(I) in the presence of other metal ions	Absorption	[136]
33	Zinc(II)	1-Methyl-1-phenyl-3-[1-hydroxyimino-2-(succinimido)ethyl]-cyclobutane (MCB)	PVC membrane	Tap water	8.0 × 10^−8^ to 1.6 × 10^−4^ mol·L^−1^	2.5 × 10^−8^ mol·L^−1^	6.0	Less selective toward Zinc(II) in the presence of Co(II) and Ni(II)	Fluorescence	[137]
34	Zinc(II)	1-(*p*-Nitrophenylazo)-2-naphthol (disperse azo dye)	PVC membrane	--	5.0 × 10^−3^ to 1.0 × 10^−6^ M	8.0 × 10^−7^	9.0	Presence of Cu(II), Co(II)and Ni(II) affect selectivity	Fluorescence	[138]
35	Zinc(II)	4-Benzoxazol-2-yl-3-hydroxyphenylallyl ether	2-Hydroxyethyl methacrylate (HEMA)	Tap river water	8.0 × 10^−5^ to 4.0 × 10^−3^ mol·L^−1^	4.5 × 10^−5^ mol·L^−1^	3.73–9.19	Good selectivityfor Zn(II) in the presence of Cd(II)	Fluorescence	[139]
36	Zinc(II)	8-Pyridylmethyloxy-2-methylquinoline	Water-soluble	Nutrition supplements/zinc gluconate solution of sanchine	7.5 × 10^−8^ to 2.5 × 10^−5^M	1.5 *×* 10^−5^ μM	4.5–9.2	Selective for Zn(II)In the presence ofother metal ions except for Cd(II)	Fluorescence	[140]
37	Zinc(II)	N-Methyl-α,β,γ,δ-tetraphenylporphine (NMTPPH)	--	Ethanol–water solution	5.0 × 10^−7^ to 1.0 × 10^−5^ mol·L^−1^	1.5 × 10^−7^ mol·L^−1^	6.3–10.5	Selective for Zn(II)affected by Cu(II) and Hg(II)	Fluorescence	[141]
38	Fe(III)	Rhodamine-based thiacalix[4]arene derivative	--	Ethanol water solution	5.0×10^−6^ to 6.0 × 10^−5^ mol·L^−1^	3.5 × 10^−8^ mol·L^−1^	6.0	Enhanced selectivity for Fe(III)	Fluorescence	[142]
39	Fe(III)	1-(D-Gluco-pyranosyl-20-deoxy-20-iminomethyl)-2-hydroxynaphthalene	--	Aqueous HEPES buffer solution	--	280 ppb	7.2	Selective for Fe(III) in buffer solution	Absorption	[143]
40	Fe(III)	Rhodamine-benzimidazole conjugate	--	--	6 × 10^−6^ to 4 × 10^−5^ M	1.5 ×10^−8^ M.	4.0–14.0	Selective to Fe(III) over other metal ions	Fluorescence	[144]
41	Fe(III)	Poly(9-aminofluorene)	--	HEPES buffer	5.0 × 10^−12^ to 7.6 × 10^−6^ M	3.7 × 10^−12^	7.0	Selective towards Fe(III)	Fluorescence	[145]
42	Cr(III)	Rhodamine-based thiacalix[4]arene derivative	PVC matrix	Ethanol water solution	4.0×10^−6^ to 1.0 × 10^−5^ mol·L^−1^	1.6 ×10^−7^mol·L^−1^	6.0	Enhanced selectivity for Cr(III)	Fluorescence	[142]
43	Ni(II)	Thiazolotriazole derivative	PVC matrix	Aqueous solution	1.0 × 10^−9^ to 4.4 × 10^−3^ M	8.5 × 10^−10^	6.0	Presence of other metal ions has minor effects on selectivity	Fluorescence	[146]
44	Ni(II)	3,7-Diamine-5-phenothiazoniom thionineacetate	Agarose membrane	Environmental water	1.0 × 10^−1^° to 1.0 × 10^−7^ mol·L^−1^	9.3 × 10^−11^ mol·L^−1^	5.8	Good selectivity towards Ni(II) in the presence of other metal ions	Absorption	[147]
45	Ni(II)	Dibutylphthalate and 2-amino-1-cyclopentenedithio-carboxylic acid	PVC membrane	Water sample	3.1 × 10^−8^ to 8.0 × 10^−3^ M	--	--	Selective towards Ni(II) with other metal ions expects Pb(II), Cd(II) and Al(III)	Absorption	[148]
46	Ni(II)	1,2-di(*o*-Salicylaldiminophenylthio)ethane (H2DSALPTE)	PVC membrane	Water sample	1.0 × 10^−5^ to 5.0 × 10^−3^ M	8.5 × 10^−6^ μM	6.0	Selective to Ni(II) but presence of Hg(II), Cu(II) effects response time	Absorption	[149]
47	UO_2_(II)	6,7,9,10,12,13,15,16,23,24,25,26 dodecahydrodibenzo[n,v][1,4,7,10,13,17,20]pentaoxadiazacyclotricosine-22,27-dione	PVC membrane	Water sample	4.3 × 10^−6^ to 2.5 × 10^−8^M	8.0 × 10^−9^ M	4.0	Stable and selective towards UO_2_(II)		[150]
48	UO_2_(II)	1-(2-Pyridylazo)-2-naphthol (PAN), tri-*n*-octyl phosphine oxide (TOPO) and sodium tetraphenylborate (Na-TPB)	PVC membrane	Water sample	1.0 × 10^−6^ to 1.50 × 10^−4^M	8.2 × 10^−7^ M.	5.5	Good selective towards UO_2_(II)	Absorption	[151]
49	UO_2_(II)	1,1′-2,2′-(1,2-Phenylene)bis(ethene-2,1-diyl)dinaphthalen-2-ol (PBED), dibutyl phthalate (DBP) and sodium tetraphenylborate (Na-TPB)	PVC membrane	Water sample	3.9 × 10^−6^ to 8.0 × 10^−5^ mol·L^−1^	9.9 × 10^−7^ mol·L^−1^	3.0	Selective to UO_2_(II) over alkali, alkaline earth, transition, and heavy metal ions	Absorption	[152]
50	UO_2_(II)	C.I. Mordant Blue 29 (Chromazurol S)/cetyl N,N,N-trimethyl ammonium bromide	Triacetyl cellulose membrane	Water sample	3.0 × 10^−7^ to 6.0 × 10^−5^mol·L^−1^	1.1 × 10^−7^ mol·L^−1^	4.5	Presence of Cu(II), Fe(III) and Th(IV) in sample effect selectivity towards UO2(II)	Absorption	[153]
51	Al(III)	*meso*-tetra(N-Methyl-4-pyridyl)porphinetetratosylate salt (TMPyP	--	Water sample	0.1 to 1.5 mM	40 nM	5.5	Excellent selectivity for Al(III) over other metal ions	Fluorescence	[154]
52	Bi(III)	Methyltrioctyl-ammonium chloride	Triacetylcellulose membrane	water samples	3.36 × 10^−6^ to 4.80 × 10^−5^ mol·L^−1^	1.02 × 10^−6^ mol·L^−1^	--	Less selective to Bi(III) in the presence of heavy metal ions	Absorption	[155]
